# Exogenous Potassium (K^+^) Positively Regulates Na^+^/H^+^ Antiport System, Carbohydrate Metabolism, and Ascorbate–Glutathione Cycle in H_2_S-Dependent Manner in NaCl-Stressed Tomato Seedling Roots

**DOI:** 10.3390/plants10050948

**Published:** 2021-05-10

**Authors:** M. Nasir Khan, Soumya Mukherjee, Asma A. Al-Huqail, Riyadh A. Basahi, Hayssam M. Ali, Bander M. A. Al-Munqedhi, Manzer H. Siddiqui, Hazem M. Kalaji

**Affiliations:** 1 Department of Biology, Faculty of Science, College of Haql, University of Tabuk, Tabuk 71491, Saudi Arabia; rbasahi@ut.edu.sa; 2 Department of Botany, Jangipur College, University of Kalyani, West Bengal 742213, India; soumobios@gmail.com; 3 Chair of Climate Change, Environmental Development and Vegetation Cover, Department of Botany and Microbiology, College of Science, King Saud University, Riyadh 11451, Saudi Arabia; aalhuquail@ksu.edu.sa (A.A.A.-H.); hayhassan@ksu.edu.sa (H.M.A.); banderalmangethi@yahoo.com (B.M.A.A.-M.); 4 Department of Plant Physiology, Institute of Biology, Warsaw University of Life Sciences SGGW, 159 Nowoursynowska 159, 02-776 Warsaw, Poland; hazem@kalaji.pl

**Keywords:** hydrogen sulfide, ionic homeostasis, Na^+^/H^+^ antiport, oxidative stress, potassium, salinity

## Abstract

Potassium (K^+^) is one of the vital macronutrients required by plants for proper growth and blossoming harvest. In addition, K^+^ also plays a decisive role in promoting tolerance to various stresses. Under stressful conditions, plants deploy their defense system through various signaling molecules, including hydrogen sulfide (H_2_S). The present investigation was carried out to unravel the role of K^+^ and H_2_S in plants under NaCl stress. The results of the study show that NaCl stress caused a reduction in K^+^ and an increase in Na^+^ content in the tomato seedling roots which coincided with a lower H^+^-ATPase activity and K^+^/Na^+^ ratio. However, application of 5 mM K^+^, in association with endogenous H_2_S, positively regulated the Na^+^/H^+^ antiport system that accelerated K+ influx and Na+ efflux, resulting in the maintenance of a higher K^+^/Na^+^ ratio. The role of K^+^ and H_2_S in the regulation of the Na^+^/H^+^ antiport system was validated by applying sodium orthovanadate (plasma membrane H^+^-ATPase inhibitor), tetraethylammonium chloride (K^+^ channel blocker), amiloride (Na^+^/H^+^ antiporter inhibitor), and hypotaurine (HT, H_2_S scavenger). Application of 5 mM K^+^ positively regulated the ascorbate–glutathione cycle and activity of antioxidant enzymes that resulted in a reduction in reactive oxygen species generation and associated damage. Under NaCl stress, K^+^ also activated carbohydrate metabolism and proline accumulation that caused improvement in osmotic tolerance and enhanced the hydration level of the stressed seedlings. However, inclusion of the H_2_S scavenger HT reversed the effect of K^+^, suggesting H_2_S-dependent functioning of K^+^ under NaCl stress. Therefore, the present findings report that K^+^, in association with H_2_S, alleviates NaCl-induced impairments by regulating the Na^+^/H^+^ antiport system, carbohydrate metabolism, and antioxidative defense system.

## 1. Introduction

Soil salinity is one of the menaces that limits crop production worldwide. Excessive accumulation of salts in the soil occurs due to poor irrigation and fertilizer management practices combined with high temperature and drought [1]. Salinity causes degradation of the soil structure and function that results in the loss of 1.5 million ha of arable land every year, which culminates in an annual loss of USD 31 million [2]. Salinity affects plants at all stages of their life cycle, from seed germination to senescence, by modulating water and nutrient uptake, photosynthesis, pigment biosynthesis, and enzyme activities [3]. The generation of osmotic stress is the hallmark of salinity, predominantly caused by an excessive accumulation of sodium (Na^+^) in the soil, that reduces the osmotic potential of soil and thus the availability of water to the plants [4]. To counter osmotic stress, plants synthesize various osmolytes, such as sugars, and amino acids (proline, glycine betaine, and taurine) which assist the plants in maintaining optimum osmotic pressure to sustain normal hydration levels of the plants [5]. Among these osmolytes, sugars or soluble carbohydrates are of substantial importance in combating osmotic stress and the maintenance of carbon storage of the plants under stressful conditions [6]. Sucrose is the most widely distributed soluble carbohydrate in plants. Sucrose is a link between source and sink tissues, serves as a major energy source under stressful environmental conditions, and acts as a storage carbohydrate in the form of starch [7].

The influx of Na+ in the root system depolarizes the plasma membrane that inhibits potassium (K^+^) influx and enhances K^+^ loss, causing cellular K^+^ deficiency. Such a disproportionate concentration of K^+^ and Na^+^ results in a lower K^+^/Na^+^ ratio, causing disturbance in ion homeostasis that creates ionic stress and damage to the cellular system and enzyme activities [3,8]. Therefore, retention of K^+^ and supplementing plants with K^+^ would be beneficial to maintain K^+^ homeostasis under NaCl stress. It is a well-known fact that K^+^ is the second most abundant nutrient in plants, K^+^ comprises 2 to 10% of plant dry weight [9], and has been supplied to crops for over 250 years. It plays a decisive role in the proper growth and development of crop plants [10]. It has been observed that over 70 enzymes exhibit sensitivity to K^+^ [11]. The involvement of K^+^ has been well investigated in the movement of leaves, opening and closing of stomata, and axial growth and tropisms [11,12]. It also assists plants in maintaining charge balance across the membranes [12]. Therefore, under NaCl stress conditions, intracellular K^+^ homeostasis is particularly important for maintaining a higher K^+^/Na^+^ ratio and for proper functioning of the cellular system [3,13]. The transport of Na^+^ and K^+^ across the plasma membrane is carried out through a membrane-associated Na^+^/H^+^ antiport system including H^+^-ATPase, K^+^ channels and transporters, and a Na^+^/H^+^ antiporter. Uptake of K^+^ in plants is probably carried out through proton-driven potassium transporters (K^+^/H^+^ symporters) which provide K^+^ influx against the concentration gradient [14]. In addition, an influx of K^+^ also occurs by K^+^ channel-mediated facilitated diffusion of K^+^ ions across biological membranes.

The formation of reactive oxygen species (ROS) is a key response of plants to any stress. At lower concentrations, these ROS take part in a signaling process that assists in activating the defense system of plants. However, long-term exposure to salinity causes overproduction of ROS that creates oxidative stress and hinders vital metabolic processes, and causes DNA damage and peroxidation of membrane lipids [3,15]. Plants deal with oxidative stress by deploying their antioxidative system. This system comprises enzymatic antioxidants viz. superoxide dismutase (SOD), peroxidase (POX), catalase (CAT), ascorbate peroxidase (APX), and glutathione reductase (GR). The antioxidant enzyme SOD acts as the front-line defender and converts superoxide (O_2_^•−^) radicals to hydrogen peroxide (H_2_O_2_). A high level of H_2_O_2_ is phytotoxic, which is controlled by POX, CAT, APX, and GR. The role of a non-enzymatic antioxidant system, namely the ascorbate–glutathione (AsA-GSH) cycle, is extremely crucial in the detoxification of H_2_O_2_ and maintenance of cellular redox homeostasis [16]. Salinity stress also induces excessive generation of methylglyoxal (MG) which adversely affects vital cellular activities and causes inhibition of the antioxidant defense system. However, plants counter detrimental effects of MG by using the enzymatic components of the glyoxalase system [3].

In spite of an array of defense systems, the protection of plants depends on the perception of a stress stimulus followed by timely and precise activation of the defense system before the inception of the damage. All these events are accomplished by the involvement of various signaling molecules which serve as a communicating network for transmitting the stress stimulus to initiate a defense response to the specific stimulus. Of these, hydrogen sulfide (H_2_S), a gaseous signaling molecule, is well known for its substantial role in the growth of plants and in the acquisition of stress tolerance [3,17,18,19]. Plants synthesize H_2_S from the degradation of L/D-cysteine in a reaction catalyzed by L-cysteine desulfhydrase (LCD) and D-cysteine desulfhydrase (DCD), respectively. Substantial evidence is available on the involvement of H_2_S in the responses of plants to salinity [3,20]. Under NaCl stress, H_2_S lowers the Na^+^ concentration and prevents salt-induced K^+^ loss which assist the plants in maintaining redox balance, ion homoeostasis, and modulation of enzyme activities [3].

Although, a significant number of studies have been carried out on the role of H_2_S in the tolerance of plants to various abiotic stresses, vague or meager information is available on the interactive role of K^+^ and H_2_S in the protection of plants against NaCl stress. The present investigation was carried out to understand the impact of K^+^ supplementation on the endogenous level of H_2_S and to comprehend their interactive role in the regulation of the Na^+^/H^+^ antiport system, carbohydrate metabolism, and antioxidative defense during plant responses to NaCl stress in tomato seedlings.

## 2. Results

### 2.1. Exogenous Potassium Supplementation Restores Tomato Plant Growth through Endogenous H_2_S Signaling under NaCl Stress

**Figure 1:**
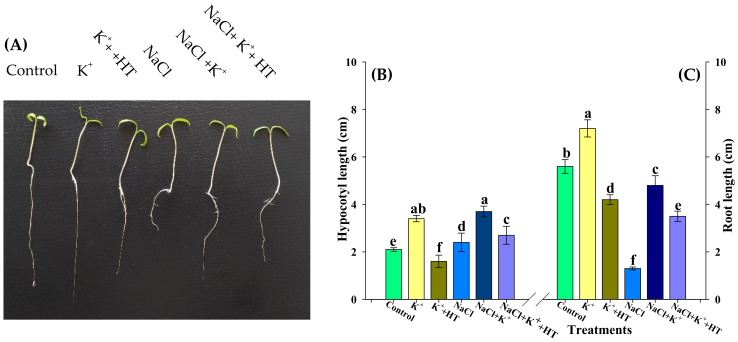
Effect of various treatments on (**A**) Phenotypic appearance, (**B**) hypocotyl length, and (**C**) root length of tomato. Double deionized water (control), 5 mM K_2_CO_3_ (K^+^), 100 mM NaCl (NaCl), 1 mM hypotaurine (HT: H_2_S scavenger). Values are means of five independent replicates, with bars indicating SE. Bars with different letters indicate that differences were statistically significant at *p* < 0.05 (DMRT).

Tomato seedlings subjected to NaCl stress (100 mM) exhibited a significant reduction in growth. However, a more prominent effect of NaCl inhibition was observed on the primary root elongation than the hypocotyl (Figure 1A–C). However, K^+^ supplementation (5 mM K_2_CO_3_) resulted in the partial recovery of NaCl stress-induced growth inhibition. This was evident from an increase in hypocotyl and primary root length. Nevertheless, exogenous K^+^ supplementation also appeared to exert similar growth-promoting effects in control seedlings. However, the effect was more pronounced in NaCl-stressed seedlings supplemented with the K^+^ treatment. Interestingly, seedlings (control and salt stressed) co-treated with K and hypotaurine (1 mM HT; endogenous H_2_S inhibitor) reveal significant differences in growth conditions, wherein HT tends to partially reverse the effect of K^+^ supplementation on hypocotyl elongation and primary root extension. This is manifested by partial reduction in hypocotyl elongation and primary root extension in the presence of K^+^ and HT treatment (Figure 1A–C).

### 2.2. Endogenous H_2_S Facilitates Exogenous Potassium-Mediated Na^+^/K^+^ Homeostasis and Elevates H^+^-ATPase Activity in Tomato Seedling Roots under NaCl Stress

**Figure 2:**
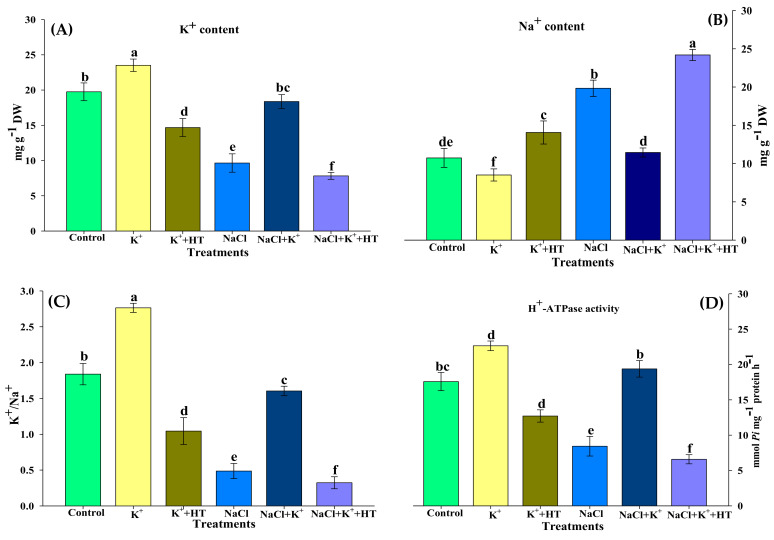
Effect of K^+^ and NaCl on K^+^ and Na^+^ concentration and H^+^-ATPase activity in the roots of tomato seedlings. (**A**) K^+^ content, (**B**) Na^+^ content, (**C**) K^+^/Na^+^ ratio, and (**D**) H^+^-ATPase activity. Double deionized water (control), 5 mM K_2_CO_3_ (K^+^), 100 mM NaCl (NaCl), 1 mM hypotaurine (HT: H_2_S scavenger). Values are means of five independent replicates, with bars indicating SE. Bars with different letters indicate that differences were statistically significant at *p* < 0.05 (DMRT).

NaCl stress (100 mM) results in a 2-fold increase in Na^+^ content in tomato seedling roots which is accompanied by a concomitant 2-fold reduction in K^+^ content (Figure 2A,B). A NaCl stress-induced alteration in Na^+^ and K^+^ content is also evident in the form of a 5.5-fold reduction in the K^+^/Na^+^ ratio (Figure 2C). H^+^-ATPase activity also appears to be negatively affected by NaCl stress, wherein it exhibits around a 2.5-fold reduction in activity in comparison with the control (Figure 2D). NaCl-stressed seedlings supplemented with exogenous K^+^ showed recovery in the K^+^/Na^+^ ratio and K^+^ content, followed by a restriction in the extent of Na^+^ accumulation (Figure 2A–C). H^+^-ATPase activity also exhibited recovery in the presence of exogenous K^+^ supplementation (Figure 2D). Interestingly, K^+^ normalized the effect of NaCl stress on Na^+^/K^+^ homeostasis and H^+^-ATPase activity. In order to assess the role of endogenous H_2_S in mediating a K^+^-induced response, seedlings were co-treated with K^+^ and HT both in the absence and presence of NaCl stress. The findings revealed that endogenous H_2_S appears to function as a facilitator of exogenous K^+^-mediated Na^+^/K^+^ homeostasis and H^+^-ATPase activity. HT treatment in the presence of K^+^ led to an alteration in Na^+^/K^+^ homeostasis (low K^+^/Na^+^ ratio) accompanied by partial inhibition of H^+^-ATPase activity (Figure 2A–D). Thus, in the presence of exogenous K^+^, endogenous H_2_S functions as a positive regulator of H^+^-ATPase activity and restricts Na^+^ accumulation.

**Figure 3:**
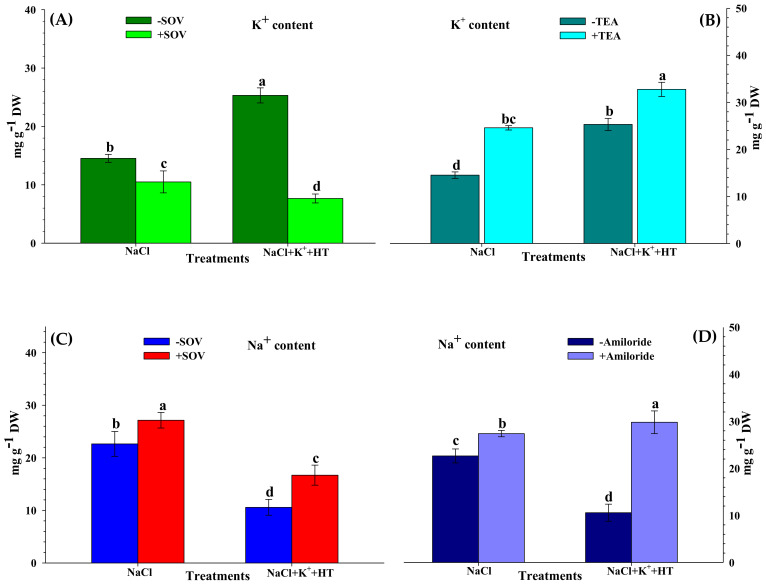
Effect of PM inhibitors on K^+^ and Na^+^ content in the presence or absence of K^+^ and HT in NaCl-stressed (48 h) tomato roots. Effect of SOV (**A**) and TEA (**B**) on K^+^ content, effect of SOV (**C**) and amiloride (**D**) on Na^+^ content. Addition of 500 µM sodium orthovanadate (SOV, PM H^+^-ATPase inhibitor), 20 mM tetraethylammonium chloride (TEA, K^+^ channel blocker), 50 µM amiloride (Na^+^/H^+^ antiporter inhibitor), 1 mM hypotaurine (HT: H_2_S scavenger), 5 mM K_2_CO_3_ (K^+^), 100 mM NaCl (NaCl). Values are means of five independent replicates, with bars indicating SE. Bars with different letters indicate that differences were statistically significant at *p* < 0.05 (DMRT).

Furthermore, to validate the role of endogenous H_2_S on the modulation of Na^+^/K^+^ homeostasis, NaCl-stressed tomato seedlings were treated with 500 µM sodium orthovanadate (SOV, PM H^+^-ATPase inhibitor), 20 mM tetraethylammonium chloride (TEA, K^+^ channel blocker), and 50 µM amiloride (Na^+^/H^+^ antiporter inhibitor) both in the absence and presence of K^+^+HT (Figure 3A–D). Treatment with SOV exhibited contrasting effects on Na^+^ and K^+^ content in the presence and absence of K+HT, wherein SOV reduced K^+^ accumulation, followed by increased Na^+^ uptake in NaCl-stressed seedling roots. The effect was more pronounced in the presence of K^+^+HT. Application of TEA positively enhanced K^+^ accumulation in the presence and absence of HT. The effect of amiloride was manifested by increased accumulation of Na^+^ in the roots of NaCl-stressed tomato seedlings, which was remarkably higher in the presence of K^+^+HT.

### 2.3. Exogenous K^+^ Supplementation Positively Upregulates H_2_S Biosynthesis in Tomato Seedling Roots

**Figure 4:**
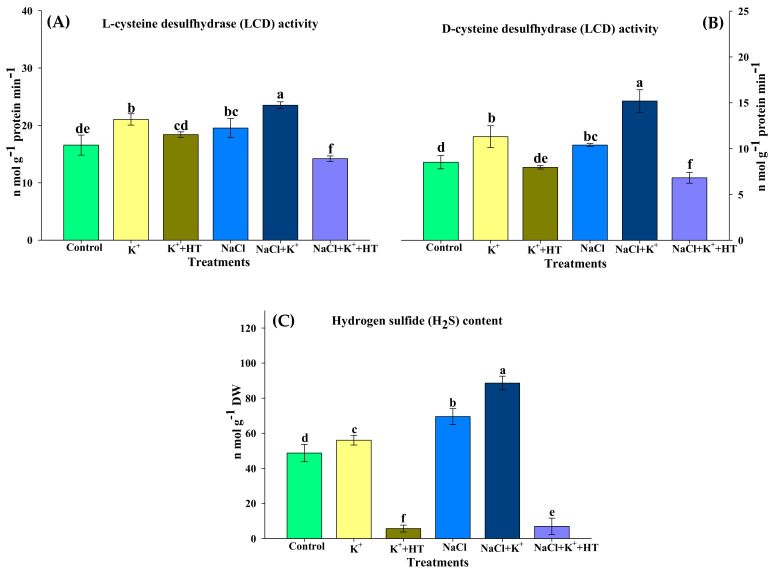
Effect of K^+^ and NaCl on the activity of H_2_S-biosynthesizing enzymes and H_2_S content in the roots of tomato seedling roots. (**A**) L-cysteine desulfhydrase (LCD) activity, (**B**) D-cysteine desulfhydrase (DCD) activity, and (**C**) hydrogen sulfide (H_2_S) content. Double deionized water (control), 5 mM K_2_CO_3_ (K^+^), 100 mM NaCl (NaCl), 1 mM hypotaurine (HT: H_2_S scavenger). Values are means of five independent replicates, with bars indicating SE. Bars with different letters indicate that differences were statistically significant at *p* < 0.05 (DMRT).

In order to investigate the effect of NaCl stress and exogenous K^+^ supplementation on endogenous H_2_S homeostasis, seedlings (control and NaCl stressed) were treated with K^+^ and HT separately and in combination (Figure 4A–C). Analysis of H_2_S-biosynthesizing enzymes (LCD and DCD) revealed that NaCl stress significantly upregulated both LCD and DCD activity, which was higher in the presence of exogenous K^+^. Elevation in the activity of LCD and DCD in the presence of NaCl stress and/or NaCl+K^+^ further coincided with an increase in endogenous H_2_S accumulation in tomato seedling roots. Roots of control seedlings raised in the absence of NaCl stress also exhibited exogenous K^+^-mediated positive regulation of H_2_S biosynthesis. Correspondingly, treatment with HT (H_2_S scavenger) reversed the effect of NaCl stress and exogenous K^+^ on LCD and DCD activity, thus reducing endogenous H_2_S accumulation. Therefore, it is evident that exogenous K^+^ positively upregulated H_2_S biosynthesis, wherein endogenous H_2_S levels function as an inducer of LCD/DCD activity both in the absence and presence of NaCl stress.

### 2.4. Exogenous K^+^ Supplementation Mediates Endogenous H_2_S-Dependent Regulation of Oxidative Stress, AsA-GSH Metabolism, and Antioxidant Enzyme Activity in Tomato Seedling Roots

In order to measure the status of oxidative stress, various parameters were analyzed in the presence and absence of NaCl stress and K^+^ and HT treatment. NaCl stress significantly increased hydrogen peroxide (H_2_O_2,_ n mol g^−1^ DW) content, superoxide (O_2_^•−^, n mol g^−1^ DW) content, thiobarbituric acid reactive substances (TBARSs, n mol g^−1^ DW), and electrolyte leakage (ELKG, %) in a range of 2- to 2.5-fold (Table 1), whereas relative water content (RWC, %) was reduced by 2-fold, thus suggesting the intensity of oxidative stress induced by 100 mM NaCl. Interestingly, application of exogenous K^+^ led to significant recovery in all the parameters of oxidative stress which was further reversed by HT treatment. Scavenging of endogenous H_2_S (HT treatment) brought about a remarkable increase in all the parameters of oxidative stress. Thus, the K^+^-mediated decrease in oxidative stress is largely facilitated by endogenous H_2_S levels. The interplay of K^+^ and H_2_S in regulating oxidative stress was also evident in control seedlings raised in the absence of NaCl stress.

**Figure 5:**
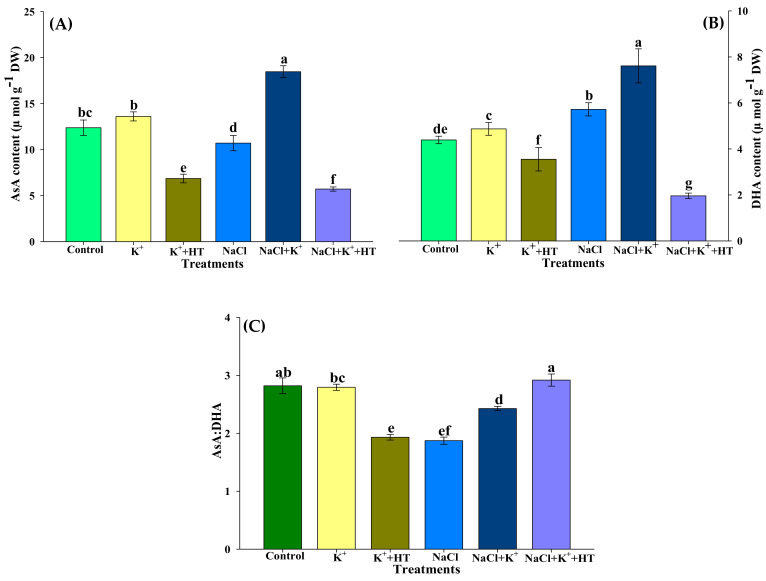
Effect of K^+^ and NaCl on the non-enzymatic components of ascorbate–glutathione cycle in tomato seedling roots. (**A**) Ascorbate (AsA) content, (**B**) dehydroascorbate (DHA) content, and (**C**) AsA:DHA ratio. Double deionized water (control), 5 mM K_2_CO_3_ (K^+^), 100 mM NaCl (NaCl), 1 mM hypotaurine (HT: H_2_S scavenger). Values are means of five independent replicates, with bars indicating SE. Bars with different letters indicate that differences were statistically significant at *p* < 0.05 (DMRT).

Exposure of seedlings to NaCl stress reduced the AsA content, which was further accompanied by a marginal increase in dehydroascorbate (DHA) content in tomato seedling roots (Figure 5A,B). Exogenous K^+^ supplementation in the presence of NaCl stress positively upregulated both AsA and DHA content, which was reversed in the presence of HT treatment. Similar trends in AsA and DHA content were recorded for K^+^ and HT treatment in control seedlings raised in the absence of NaCl stress. Interestingly, the AsA:DHA ratio exhibited differential effects in the presence and absence of NaCl stress and K^+^ and HT treatment, where K^+^ and HT reduced the AsA:DHA ratio in control seedlings. Contrastingly, the AsA:DHA ratio was increased in the presence of K^+^+HT in NaCl-stressed seedlings. Modulation of APX activity (Figure 7A) also coincided with increased AsA content (Figure 5A) in the presence/absence of NaCl stress and/or exogenous K^+^ treatment.

**Figure 6:**
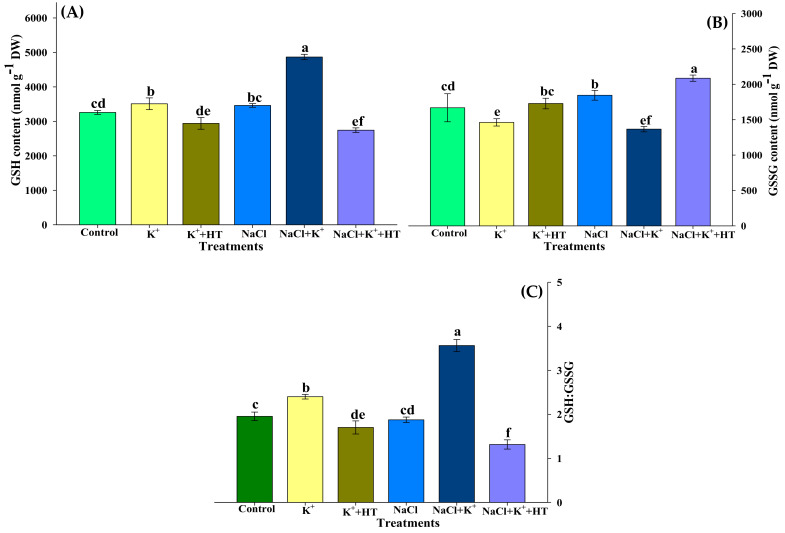
Effect of K^+^ and NaCl on the non-enzymatic components of ascorbate–glutathione cycle in tomato seedling roots. (**A**) Reduced glutathione (GSH) content, (**B**) oxidized glutathione (GSSG) content, and (**C**) GSH:GSSG ratio. Double deionized water (control), 5 mM K_2_CO_3_ (K^+^), 100 mM NaCl (NaCl), 1 mM hypotaurine (HT: H_2_S scavenger). Values are means of five independent replicates, with bars indicating SE. Bars with different letters indicate that differences were statistically significant at *p* < 0.05 (DMRT).

**Figure 7:**
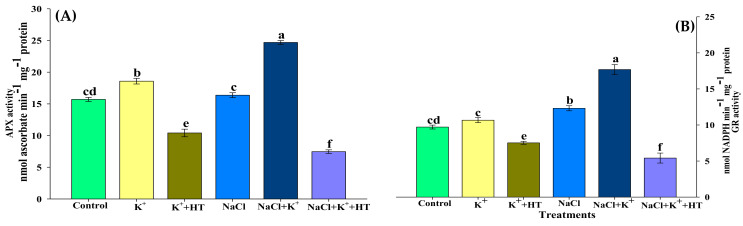
Effect of K^+^ and NaCl on the enzymatic components of ascorbate–glutathione cycle in tomato seedling roots. (**A**) Ascorbate peroxidase (APX) activity and (**B**) glutathione reductase (GR) activity. Double deionized water (control), 5 mM K_2_CO_3_ (K^+^), 100 mM NaCl (NaCl), 1 mM hypotaurine (HT: H_2_S scavenger). Values are means of five independent replicates, with bars indicating SE. Bars with different letters indicate that differences were statistically significant at *p* < 0.05 (DMRT).

Reduced (GSH) and oxidized (GSSG) forms of glutathione were analyzed in the control and NaCl-stressed seedling roots treated with K^+^ and/or HT (Figure 6A,B). NaCl stress marginally increased the GSH and GSSG ratio in roots. However, supplementation of K^+^ in NaCl-stressed seedling roots increased the GSH content, accompanied by a concomitant reduction in GSSG levels (Figure 6A,B). Application of HT treatment reversed the effects of K^+^ on GSH and GSSG content. Furthermore, similar observations were recorded in control seedling roots which revealed that endogenous H_2_S levels are crucial for enhanced GSH formation in the presence of exogenous K^+^ supplementation both in the absence and presence of NaCl stress. Exogenous K^+^ increased the GSH:GSSG ratio in both control and NaCl-stressed seedling roots which was then reversed upon HT treatment (Figure 6C). Enhanced accumulation of GSH (Figure 6A) correlated with elevation in GR activity (Figure 7B) in the presence/absence of NaCl stress and/or exogenous K^+^ treatment. The malleability of the findings for the H_2_S-dependent regulation of AsA–GSH metabolism was affirmed by the application of HT treatment which could partially reverse the effect of the K^+^-mediated response.

**Figure 8:**
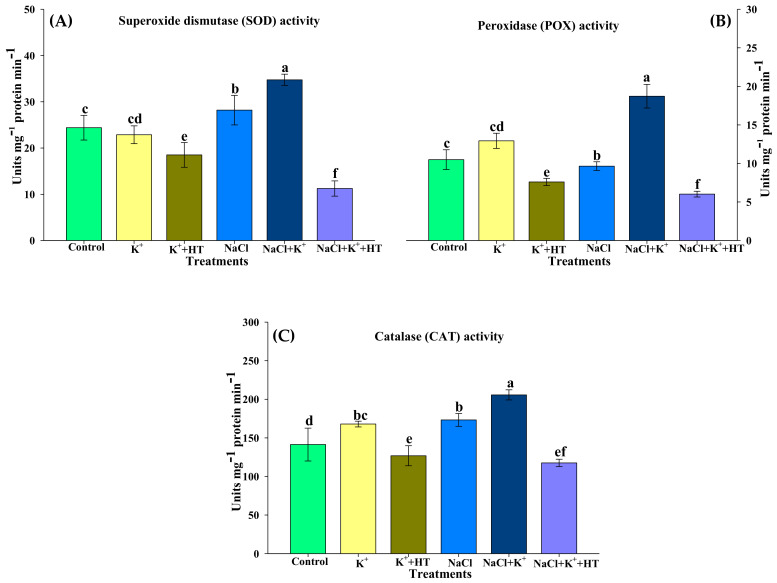
Effect of K^+^ and NaCl on the activity of enzymes involved in maintaining reactive oxygen species homeostasis in tomato seedling roots. (**A**) Superoxide dismutase (SOD) activity, (**B**) peroxidase (POX) activity, and (**C**) catalase (CAT) activity. Double deionized water (control), 5 mM K_2_CO_3_ (K^+^), 100 mM NaCl (NaCl), 1 mM hypotaurine (HT: H_2_S scavenger). Values are means of five independent replicates, with bars indicating SE. Bars with different letters indicate that differences were statistically significant at *p* < 0.05 (DMRT).

Under NaCl stress, SOD, POX, and CAT activity exhibited significant elevation which was higher in the presence of exogenous K^+^ supplementation (Figure 8A–C). Exogenous K^+^ supplementation marginally reduced SOD activity in control seedlings (Figure 8A). Activity of POX was observed to exhibit no significant increase in the presence of NaCl stress, which, however, was also positively upregulated in the presence of K^+^ supplementation (Figure 8B), whereas application of K^+^ to stressed seedlings caused a substantial increase in the activity of SOD and POX. A similar trend was also observed with the activity of CAT (Figure 8C). However, the effect of K^+^ on the activity of theses enzymes was abolished by the application of endogenous H_2_S scavenger HT. This signifies that all three antioxidant enzymes exhibited endogenous H_2_S-dependent regulation during exogenous K^+^ supplementation.

### 2.5. Exogenous K^+^ Supplementation Mediates MG Catabolism and Enhances Pro Content through Endogenous H_2_S Signaling

The presence of NaCl in the growth medium significantly upregulated Gly I and Gly II activity in tomato seedling roots which was accompanied by an increase in MG content (Table 2). However, K^+^ supplementation reduced the MG content and was associated with a further enhancement in Gly I and Gly II activity. Exogenous K^+^ also functioned as a positive regulator of the activity of these two enzymes in the control seedling roots. K^+^-mediated modulation of Gly I and Gly II activity and MG content exhibited partial reversal in the presence of HT treatment (Table 2). Thus, MG catabolism is enhanced by the activity of Gly I and Gly II in the presence of K^+^ supplementation, wherein endogenous H_2_S levels are crucial to mediate the effects.

A significant increase in Pro accumulation was observed in the presence of NaCl stress which was further instigated by K^+^ supplementation (Table 2). Control seedlings did not exhibit any significant changes in Pro content in response to K^+^ supplementation. However, the endogenous H_2_S inhibitor HT reduced Pro levels both in control and NaCl-stressed seedlings.

### 2.6. Endogenous H_2_S Levels Are Crucial for K^+^-Mediated Modulation of Carbohydrate Metabolism in Tomato Seedling Roots under NaCl Stress

**Figure 9:**
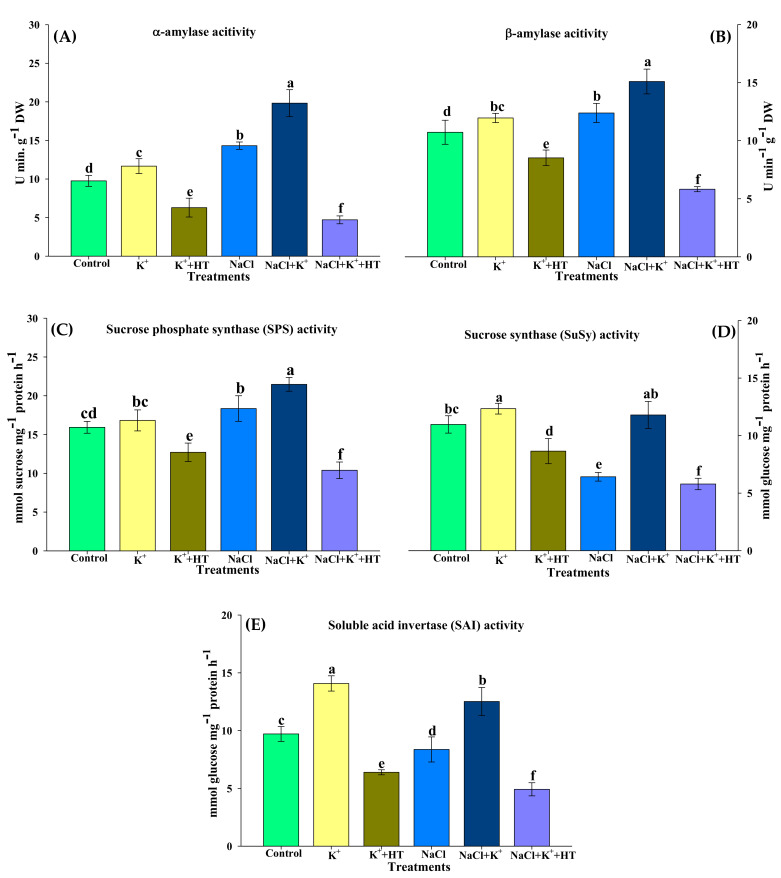
Effect of K^+^ and NaCl on the activity of enzymes inolved in carbohydrate metabolism. (**A**) α-amylase activity, (**B**) β-amylase activity, (**C**) sucrose phosphate synthase (SPS) activity, (**D**) sucrose synthase (SuSy) activity, and (**E**) soluble acid invertase (SAI) activity in tomato roots. Double deionized water (control), 5 mM K_2_CO_3_ (K^+^), 100 mM NaCl (NaCl), 1 mM hypotaurine (HT: H_2_S scavenger). Values are means of five independent replicates, with bars indicating SE. Bars with different letters indicate that differences were statistically significant at *p* < 0.05 (DMRT).

**Figure 10:**
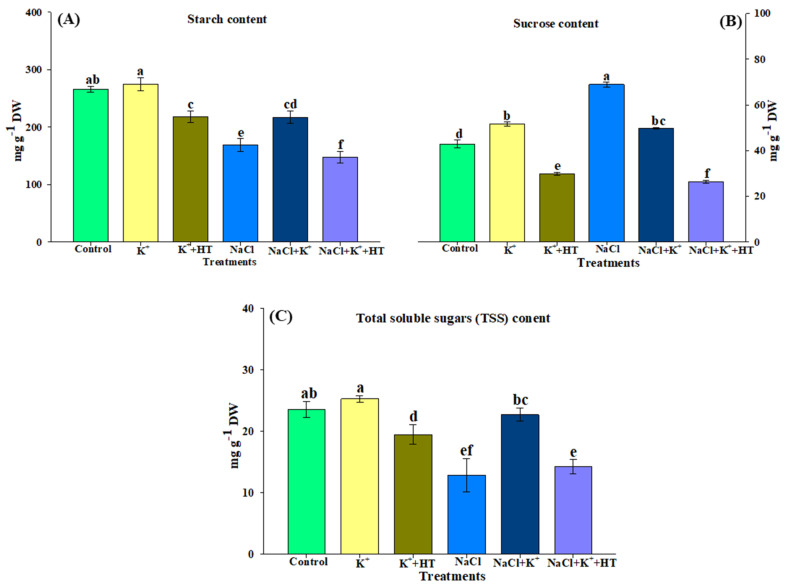
Effect of K^+^ and NaCl on the concentration of carbohydrates in tomato roots. (**A**) Starch content, (**B**) sucrose content, and (**C**) total soluble sugar (TSS) content in tomato roots. Double deionized water (control), 5 mM K_2_CO_3_ (K^+^), 100 mM NaCl (NaCl), 1 mM hypotaurine (HT: H_2_S scavenger). Values are means of five independent replicates, with bars indicating SE. Bars with different letters indicate that differences were statistically significant at *p* < 0.05 (DMRT).

The effect of exogenous K^+^ and endogenous H_2_S on sucrose and starch metabolism was analyzed by measuring α-amylase, β-amylase, SPS, SuSy, and SAI activity and starch–sugar content (Figure 9A–E and Figure 10A–C). NaCl stress marginally increased α-amylase, β-amylase, and SPS activity, while SuSy and SAI activity exhibited a significant reduction (Figure 9A–E). This was accompanied by a remarkable increase in sucrose content (Figure 8B). However, K^+^ supplementation during NaCl stress elevated α-amylase, β-amylase, SPS, and SuSy activity, followed by a reduction in sucrose content. In the control seedling roots, exogenous K^+^ did not exhibit any remarkable effects on SPS–SuSy activity or sucrose content. The K^+^-mediated effects on sucrose metabolism were established to occur through endogenous H_2_S levels, as was evident from HT treatment.

Activity of SAI exhibited K^+^-mediated upregulation both in the absence and presence of NaCl stress. NaCl stress downregulated SAI activity in tomato seedling roots (Figure 9E). Starch and TSS content also exhibited a reduction in the presence of NaCl stress which, however, increased upon K^+^ supplementation. HT treatment caused partial reversal of the effects of the K^+^-mediated increase in starch and TSS content (Figure 10A,C). Sucrose content showed an increase under NaCl stress, which, however, was decreased when stressed plants were supplemented with K^+^ (Figure 10B).

## 3. Discussion

Potassium is one among the various essential macronutrients and is known to regulate a myriad of physiological and biochemical events in plant cells. Potassium functions as an important regulator of more than fifty metabolic enzymes and is also involved in osmotic adjustment, chlorophyll biosynthesis, and nitrogen metabolism in plants [13,21,22]. Ionic stress is known to alter the efficiency of K^+^ influx within root cells [23,24]. Thus, osmotic tolerance during NaCl stress is crucially regulated by intracellular K^+^ homeostasis which also provides stress resilience by modulating various metabolic pathways in cells [3,13,19]. Despite the wide range of information available on the role of K^+^ and H_2_S as stress regulatory biomolecules in plants, not much information is available on the crosstalk of H_2_S and K^+^. Recent investigations have reported the crosstalk events associated with H_2_S, secondary messengers, and phytohormones [3,19,20,25].

In view of understanding the role of endogenous H_2_S signaling in exogenous K^+^-mediated alleviation of NaCl stress, the present investigations were performed in tomato seedling roots (NaCl stressed) treated with exogenous K^+^ and/or HT (endogenous H_2_S scavenger). The present findings reveal that exogenous K^+^ functions as a positive regulatory molecule associated with growth normalization and redox homeostasis and alleviates NaCl-stressed alterations in sugar metabolism. However, these results raise the question of how seedling phenotypes (growth reduction) might arise when deprived of nutrient medium during various treatments. Thus, further investigations are necessary to answer the question in detail. K^+^ deficiency during NaCl stress is likely to disrupt the function of various metabolic enzymes [13]. Earlier reports from an author’s laboratory revealed interplay between Ca^2+^ and ROS in K-deficient NaCl-stressed conditions [3].

### 3.1. K^+^-Instigated Synthesis of H_2_S Mediates Growth Normalization, Na^+^/H^+^ Antiport Activity, and Ion Homeostasis in Tomato Seedling Roots under NaCl Stress

The present work demonstrates the role of K^+^ in instigating endogenous H_2_S accumulation, which is in good agreement with the elevated activity of LCD and DCD in NaCl-stressed tomato seedling roots (Figure 4A–C). LCD activity and endogenous H_2_S content have been known to impart salt stress tolerance and reduce electrolytic leakage in *Solanum lycopersicum* [20]. In the present work, endogenous H_2_S accumulation is facilitated by exogenous K^+^ supplementation which further provides resilience to NaCl stress in tomato seedlings. Endogenous H_2_S levels are often known to be indicative of the effects of abiotic stress, wherein they exhibit an increase up to 2.5-fold in comparison with non-stress conditions [19]. Thus, although NaCl stress elevates the biosynthesis of H_2_S, exogenous K^+^ supplementation further instigates endogenous H_2_S accumulation, mediated by LCD and DCD activity. Interestingly, K^+^ exerts similar effects to H_2_S metabolism both in the absence and presence of NaCl stress.

**Figure 11:**
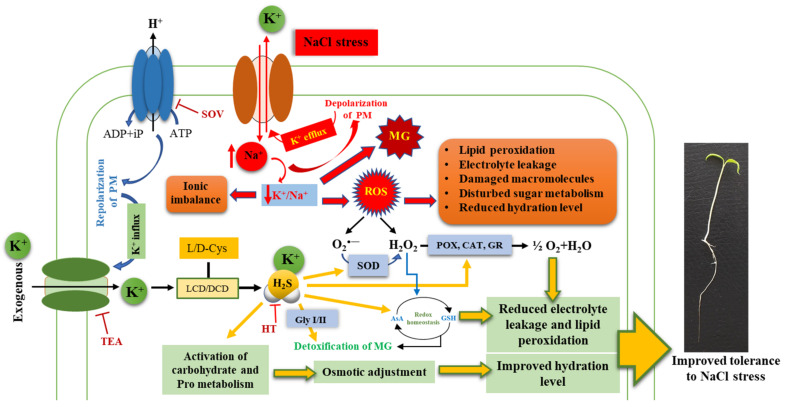
Illustration showing the role of potassium (K^+^) and hydrogen sulfide (H_2_S) in the regulation of carbohydrate metabolism and antioxidative defense system under NaCl stress. CAT: Catalase, DCD: D-cysteine desulfhydrase, Gly I/II: Clyoxalase I/II, GR: Glutathione reductase, HT: Hypotaurine, LCD: L-cysteine desulfhydrase, L/D-Cys: L/D-cysteine, MG: Methylglyoxal content, ROS: Reactive oxygen species, SOV: Sodium orthovanadate (H^+^-ATPase inhibitor), SOD: Superoxide dismutase, POX: Peroxidase, TEA: Tetraethylammonium chloride (K^+^ channel blocker).

Under NaCl stress, an increase in Na^+^ influx followed by depolarization of the plasma membrane results in K^+^ channel-activated K^+^ loss [23] that ultimately results in a lower K^+^ level and K^+^/Na^+^ ratio (Figure 2A–C), which disturbs ion homeostasis and causes cell death [26]. These adverse effects of NaCl on tomato seedling growth manifested in the reduction of hypocotyl length and primary root elongation. Tomato plants subjected to NaCl stress exhibit reduced dry weight, enhanced ELKG, and inhibition of plant growth [20]. K^+^ supplementation [3,19,20] (present work) results in the recovery of plant growth which is further reversed in the presence of HT (Figure 1A–C). H_2_S has been known to be a positive regulator of plant growth during abiotic stress [3,17,19,20,27]. Similarly, in the present work, HT-induced reversal of K^+^-mediated growth recovery indicates that endogenous H_2_S levels are crucial for K^+^-assisted growth of tomato plants during NaCl stress. A K^+^-mediated improvement in seedling growth coincides with improved K^+^/Na^+^ homeostasis (Figure 2C) and increased RWC (Table 1). Salinity stress is known to modulate K^+^ transport across roots, thus normalizing ionic balance [24]. The regulation of polarized membrane potential determines salinity tolerance capacity of the plant by keeping a higher intracellular K^+^ concentration and K^+^/Na^+^ ratio [28]. The membrane polarization is maintained by active H^+^ pumping or by low energy-consuming K^+^ efflux [24]. Under NaCl stress, the activity of H^+^-ATPase was reduced (Figure 2D) along with a decrease in K^+^ content (Figure 2A), implying that under NaCl stress, the seedlings might have tried to reverse membrane depolarization through a K^+^ efflux mechanism instead through energy-consuming H^+^ pumping [24]. It has been suggested that the K^+^ efflux may act as a ‘metabolic switch’ that inhibits metabolic activities to conserve energy for defense and reverse NaCl-induced damage [29]. Therefore, a decrease in TSS content (Figure 10C) is suggestive of a K^+^ deficiency-induced reduction in primary metabolic processes (Figure 11).

However, exogenous K^+^ tends to normalize H^+^-ATPase activity [30] which creates a H^+^ gradient across the membrane that causes repolarization of the plasma membrane (Figure 11). A repolarized membrane induced secondary active mechanisms (K^+^ transporters or H^+^/K^+^ symporters) that accelerated K^+^ influx and Na^+^ efflux (Figure 11), leading to a higher K^+^/Na^+^ ratio in K^+^-supplemented plants (Figure 2C). Therefore, exogenous K^+^ supplementation essentially regulates secondary efflux mechanisms and restricts Na^+^ accumulation in cells [3,24]. Thus, improvement in plant growth indicates less Na^+^ accumulation and improved water potential in tomato seedlings subjected to K^+^ supplementation. Moreover, a K^+^-induced increase in the accumulation of TSS content (Figure 10C) might have fulfilled the energy requirement of the ion transport mechanism. However, the addition of HT caused a reversal of the K^+^ effects which signifies H_2_S-dependent functioning of K^+^ under NaCl stress. Furthermore, evidence for an alteration in K^+^ and Na^+^ content in the presence of treatment with SOV (PM H^+^-ATPase inhibitor), TEA (K^+^ channel blocker), and amiloride (Na^+^/H^+^ antiporter inhibitor) in the presence or absence of NaCl+K^+^+HT revealed that endogenous H_2_S facilitates K^+^-mediated secondary active ion transport mechanisms in tomato seedling roots. H_2_S is known to exert positive effects on gene regulation and phosphorylation-mediated upregulation of plasma membrane-bound H^+^-ATPase activity [31]. Therefore, endogenous H_2_S-mediated regulation of K^+^ retention and ion homeostasis [27] indicates that in the present work, exogenous K^+^ and endogenous H_2_S appear to be beneficial in H^+^-ATPase-energized regulation of Na^+^/K^+^ homeostasis. Thus, in congruence with earlier findings from an author’s laboratory, it is postulated that endogenous H_2_S regulates K^+^ uptake and upregulates the K^+^/Na^+^ antiport system, energized by H^+^-ATPase activity [3].

### 3.2. Exogenous K^+^-Mediated Regulation of Antioxidative Defense Operates in a H_2_S-Dependent Manner

Increased ROS homeostasis is a primary mechanism for tolerance to oxidative stress [15]. In the present work, various oxidative stress parameters were analyzed in the presence and absence of NaCl stress, K^+^ and HT. The interplay of exogenous K^+^ and endogenous H_2_S was manifested by reducing the severity of oxidative stress under NaCl stress. The variation in H_2_O_2_ and O_2_^•−^ content during various treatments correlates with the modulation of antioxidant enzyme activity. The AsA–GSH metabolism indicates the extent of NaCl stress which induces an oxidative environment in the cell [3,18,19,32]. The maintenance of the GSH pool under K^+^ supplementation is indicative of normal functioning of the AsA–GSH cycle which invariably maintains a higher AsA:DHA and GSH:GSSG ratio (Figure 5A–C and Figure 6A–C), thus facilitating the maintenance of redox homeostasis [19,33]. Increased levels of LCD/DCD activity and H_2_S content during K^+^ supplementation indicate their possible role in glutathione-mediated antioxidative defense [32,34]. K^+^-induced elevation in the AsA:DHA and GSH:GSSG ratio aligns well with the increase in the activity of antioxidant enzymes, namely APX, GR, SOD, POX, and CAT (Figure 7A,B and Figure 8A–C). GSH and H_2_S are likely to exert a synergistic role in imparting NaCl stress tolerance [34]. Here, we report that exogenous K^+^-mediated endogenous H_2_S levels and GR activity under NaCl stress cause modulation of GSH content. Earlier evidence suggests the positive effect of GSH in upregulating LCD activity and H_2_S biosynthesis in salt-stressed pepper plants [34]. In the present work, a K^+^-induced surge in GSH levels is in good agreement with enhanced LCD/DCD activity and higher accumulation of H_2_S in tomato seedling roots under NaCl stress. Increased APX activity (Figure 7A) also coincides with higher AsA content (Figure 5A) in the tomato seedling roots subjected to NaCl and/or NaCl+K^+^. APX activity is also known to be positively modulated by H_2_S-mediated persulfidation [35]. APX is crucial for H_2_O_2_ detoxification in the initial stages of the AsA–GSH cycle. Furthermore, GSH-mediated modulation of antioxidative enzymes [36,37] might also result in upregulated SOD, POX, CAT, APX, and GR activity in tomato seedling roots under NaCl stress. SOD effectively catalyzes the dismutation of O_2_^•−^ to form H_2_O_2_ which is further oxidized by the activity of CAT and POX, as shown by reduced H_2_O_2_ content, TBARSs, and ELKG (Table 1). Thus, in the present work, K^+^ supplementation upregulates H_2_S-mediated antioxidative defense that assisted the plants in reducing NaCl-induced oxidative stress and associated impairments in tomato seedling roots. The present findings are in good agreement with the role of H_2_S in the modulation of antioxidant enzymes and regulation of the AsA–GSH cycle [3,17,19,38]. Although ABA signaling in guard cells promotes H_2_S-mediated persulfidation of NADPH oxidases and a subsequent increase in ROS generation [39], H_2_S is also known to reduce oxidative stress by increasing the enzyme activities of the AsA–GSH pathway [39,40].

### 3.3. Endogenous H_2_S Facilitates K^+^-Mediated Regulation of Glyoxylate Metabolism and Proline Accumulation

Methylglyoxal (MG) is a reactive signaling molecule which is prone to reactive conversion, thus leading to oxidative stress in plants. However, the glyoxalase system, which effectively comprises Gly I and Gly II, can detoxify MG into non-toxic compounds [36,41]. Thus, in addition to antioxidative defense, instigation of the glyoxalase system enables stress tolerance by avoiding oxidative stress. NaCl stress is likely to enhance MG content, as reported in tomato and mung bean [3,41,42]. Recent investigations reveal the interactive signaling role of H_2_S and MG in imparting thermotolerance in maize seedlings [43]. In the present work, NaCl stress significantly enhances MG content, wherein application of exogenous K^+^ exerts positive effects by upregulating Gly I and II activity, thus leading to a subsequent reduction in MG content (Table 2). In addition to H_2_S-mediated upregulation of the glyoxalase system [3], the present findings corroborate the fact that exogenous K^+^-mediated modulation of the glyoxalase system is positively mediated by endogenous H_2_S signals (Table 2). HT application is observed to significantly reduce the activity of Gly I and II, thus suggesting the interactive role of K^+^ and endogenous H_2_S in the regulation of MG content in tomato seedling roots under NaCl stress. Furthermore, enhanced accumulation of GSH (present work) is also likely to trigger Gly I and II activity, wherein GSH is used as a substrate for glyoxalase activity, thus reducing MG content in seedling roots [44,45].

Proline (Pro) content exhibits a remarkable increase in the presence of NaCl stress which is furthermore enhanced in the presence of exogenous K^+^ supplementation (Table 2). Abiotic stress is likely to enhance the accumulation of various compatible solutes in plants, including Pro [46]. NaCl stress causes K^+^ deficiency, thus leading to a higher accumulation of Pro in cells [47]. In line with earlier investigations (Ca^2+^ and H_2_S) from an author’s laboratory [3], the present evidence reveals the beneficial role of K^+^ and H_2_S in mediating Pro accumulation in tomato seedling roots under NaCl stress. Accumulation of Pro in the tomato seedlings is therefore likely to be associated with the maintenance of cellular pH, redox homeostasis, osmotic balance, and cellular integrity [3,48]. Our present findings are well aligned with previous investigations, wherein application of an endogenous H_2_S scavenger (HT) downregulated Pro biosynthesis in NaCl-stressed mung bean roots [3].

### 3.4. K^+^ and Endogenous H_2_S Modulate Carbohydrate Metabolism under NaCl Stress

Various pathways of carbohydrate metabolism are likely to encounter modulation during various abiotic stresses in plants [49,50]. Starch–sugar interconversion in source organs is a vital requirement for abiotic stress tolerance in plants [50,51]. In the present work, exogenous K^+^ partially enhances starch accumulation in NaCl-stressed seedling roots (Figure 10A), thus modulating starch biosynthesis and/or its accumulation [52]. An exogenous K^+^-mediated increase in starch content aligns with a partial decrease in sucrose levels in NaCl-stressed tomato seedling roots (Figure 10B). Furthermore, elevated invertase activity (Figure 9E) and higher accumulation of TSS (Figure 10C) indicate enhanced sucrose metabolism, thus leading to the formation of hexose sugars during K^+^ supplementation under NaCl stress. Although not much information is available on the role of H_2_S in the modulation of sugar metabolism in plants, Jiang et al. [53] provide a proteomic analysis of H_2_S-responsive proteins, wherein various enzymes of carbohydrate metabolism are modulated in NaCl-stressed cucumber leaves. Modulation of α-amylase and β-amylase activity (Figure 9A,B) during NaCl stress and K^+^ and HT treatment indicates the regulation of starch degradation, mediated by endogenous H_2_S signaling. SPS and SuSy are crucial as sucrose-cleaving enzymes during abiotic stress in plants [54,55]. Earlier known evidence is congruent with our present observations where exogenous K^+^ enhances SPS and SuSy activity in NaCl-stressed tomato seedling roots [21]. Furthermore, for annual plants like tomato, roots function as the major sink organ where accumulation of hexose sugar pertains to improved osmotic tolerance during NaCl stress, as shown by improved RWC (Table 1). Thus, NaCl stress is likely to modulate the response of starch–sugar interconversion and sucrose breakdown in tomato seedling roots [50,51]. The present findings provide substantial evidence on the interplay between exogenous K^+^ and endogenous H_2_S in regulating sugar metabolism during plant adaptive responses to NaCl stress. However, these results raise the question of what the mechanism of salt stress regulation in shoots of tomato seedlings is. Thus, further investigations are necessary to answer the question in detail, wherein a metabolomic approach or organic polar primary metabolite analysis of shoots could provide a clear understanding of the relationship between K^+^ and H_2_S and their impact on carbon metabolism.

## 4. Materials and Methods

### 4.1. Seed Germination and Treatments

The seeds of tomato (*Solanum lycopersicum* L. Mill.), sterilized with sodium hypochlorite solution (10%) for 10 min, were allowed to germinate in filter paper-lined Petri dishes. The seeds were supplied with Raukura’s nutrient solution and were kept in the dark at an average day/night temperature of 27/13 ± 2 °C. The nutrient solution comprised (i) macronutrient stock solution (g L^−1^): 4.94 Mg (NO_3_)_2_6H_2_O; 8.48 NH_4_NO_3_; 2.28 KNO^3^, (ii) macronutrient stock solution (g L^−1^): 2.67 KH_2_PO_4_; 1.64 K_2_HPO_4_; 6.62 K_2_SO_4_; 0.60 Na_2_SO_4_; 0.33 NaCl, and micronutrient supplement (mg L^−1^): 128.80 H_3_BO_3_; 4.84 CuCl_2_.2H_2_O; 81.10 MnCl_2_.4H_2_O; 0.83 (NH_4_)_6_ Mo_7_O_24_.4H_2_O; 23.45 ZnCl_2_; 809.84 C_6_H_15_FeO_12_. The solution was prepared by mixing 50 mL of each of the macronutrient stock solutions with 25 mL of the micronutrient supplement, and the final volume was increased to 2.25 L by diluting with DDW. The seeds were considered as germinated when the radicle reached a length of 2 mm. The germinated seeds were transferred to new filter paper-lined Petri dishes containing: (1) double deionized water (DDW; control), (2) 5 mM K_2_CO_3_ (5 mM K^+^), (3) K^+^+1 mM hypotaurine (K^+^+HT), (4) 100 mM NaCl (NaCl), (5) NaCl+K^+^, and (6) NaCl+K^+^+HT. The seedlings were treated for 10 days and each treatment was replicated five times, and hypotaurine (HT) was used as a H_2_S scavenger. After 10 days of treatment, the seedling roots were used for the estimation of various characteristics.

The role of K^+^ and H_2_S in the mechanism of K^+^ and Na^+^ transport was tested by treating the seedlings with plasma membrane (PM) inhibitors viz. 20 mM tetraethylammonium chloride (TEA, K^+^ channel blocker), 500 µM sodium orthovanadate (SOV, PM H^+^-ATPase inhibitor), and 50 µM amiloride (Na^+^/H^+^ antiporter inhibitor). The 10-day-old seedlings raised with NaCl and NaCl+K^+^+HT were treated with the inhibitors (TEA, SOV, and amiloride) for 30 min [56]. After 30 min, the concentration of K^+^ and Na^+^ was estimated in the roots of the tomato seedlings (Figure 3A–D).

### 4.2. Preparation of Crude Enzyme Extract

The roots (0.5 g) of treated tomato seedlings were used to prepare a crude enzyme extract for the assay of enzyme activity. Quantification of protein was carried out according to Bradford [57] using bovine serum albumin (BSA) as a standard.

### 4.3. Estimation of K^+^ and Na^+^ Content and Activity of Plasma Membrane H^+^-ATPase

Concentration of K^+^ and Na^+^ was examined using a flame photometer. The root tissues were dried for 72 h at 75 °C to prepare a fine powder. Root powder (1 g) was placed in a furnace at 500 °C to obtain ash which was dissolved in 5 mL 20% HCl. The solution was diluted to 50 mL using DDW and was used for the estimation of K^+^ and Na^+^ content.

Plasma membrane H^+^-ATPase (PM H^+^-ATPase; EC 3.6.1.35) activity was estimated according to Hejl and Koster [58] with some modifications [59]. The enzyme activity of PM H^+^-ATPase was assessed by measuring the production of inorganic phosphate at 700 nm.

### 4.4. Estimation of H_2_S Content and Activity of Its Biosynthesizing Enzymes

The method of Li [60] was used for the quantification of H_2_S in the roots of tomato seedlings. The activity of LCD (EC 4.4.1.1) and DCD (EC 4.4.1.15) was estimated by the method of Bloem et al. [61] and Riemenschneider et al. [62], respectively.

### 4.5. Estimation of the Components of AsA–GSH Cycle

The effect of various treatments on ascorbate (AsA) and dehydroascorbate (DHA) content was measured by the method of Takahama and Oniki [63]. The level of reduced glutathione (GSH) and oxidized glutathione (GSSG) was estimated by the method of Yu et al. [64]. The ratio of AsA:DHA and GSH:GSSG was also calculated. Activity of APX (EC 1.11.1.11) and GR (EC 1.6.4.2) was measured by the method of Nakano and Asada [65] and Foyer and Halliwell [66], respectively.

### 4.6. Assay of Antioxidant Enzymes

Activity of SOD (EC 1.15.1.1), POX (EC 1.11.1.7), and CAT (EC 1.11.1.6) was estimated according to Upadhyaya et al. [67], Beauchamp and Fridovich [68], and Cakmak and Marschner [69], respectively.

### 4.7. Estimation of MG Content, Activity of Gly I and Gly II, and Proline (Pro) Content

The concentration of methylglyoxal (MG) was estimated by the method of Wild et al. [70]. The supernatant from fresh root tissues (0.5 g) was collected and absorbance was recorded at 288 nm. MG content was calculated using a standard curve of known concentrations of MG. Activity of glyoxalase I (Gly I, EC: 4.4.1.5) and glyoxalase II (Gly II, EC: 3.1.2.6) was determined by the method of Hossain et al. [71] and Mostofa and Fujita [72], respectively. The quantification of Pro was carried out by adopting the method of Bates et al. [73].

### 4.8. Estimation of the Activity of the Enzymes Involved in Carbohydrate Metabolism

Activity of α-amylase (EC 3.2.1.1) and β-amylase (EC 3.2.1.2) was measured by the method of Tárrago and Nicolás [74] as described by Kishorekumar et al. [75]. Assay of sucrose phosphate synthase (SPS; EC 2.4.1.14), sucrose synthase (SuSy; EC 2.4.1.13), and soluble acid invertase (SAI) was performed according to Kalwade and Devarumath [76].

### 4.9. Estimation of Starch, Sucrose, and Total Soluble Carbohydrates

Starch content was estimated spectrophotometrically at 620 nm by adopting the method described by Kuai et al. [77], whereas soluble sugar and sucrose content were determined at 620 nm and 480 nm, respectively, by following the modified method of Xu et al. [78].

### 4.10. Estimation of Oxidative Stress Markers

The effect of various treatments on oxidative stress was assessed by estimating the level of H_2_O_2_ and O_2_^•−^ according to the method of Elstner and Heupel [79] and Velikova et al. [80], respectively.

The effect of treatments on oxidative stress-induced damage was evaluated by estimating lipid peroxidation by measuring thiobarbituric acid reactive substances (TBARSs) according to Cakmak and Horst [81]. Relative water content (RWC) was measured according to Yamasaki and Dillenburg [82] using fresh weight, dry weight, and turgid weight of the roots [83]. Electrolyte leakage (ELKG) was determined according to Lutts et al. [84]. The detailed method is described by Khan et al. [83].

### 4.11. Data Analysis

Statistical analysis of the data was performed by one-way analysis of variance (ANOVA). The values were expressed as means ± standard error of five independent replicates. Differences between treatment means were compared statistically using Duncan’s multiple range test (DMRT) at *p* < 0.05 by SPSS Ver. 20 statistical software (SPSS Inc., Chicago, IL, USA).

## 5. Conclusions

The present evidence affirms that exogenous K^+^ is beneficial in imparting NaCl stress tolerance in tomato seedlings, wherein an endogenous H_2_S signal is involved in mediating various responses associated with physiological and biochemical attributes in the plant. Regeneration of the redox milieu and ion homeostasis by K^+^ and H_2_S likely results in partial normalization of growth in NaCl-stressed tomato seedlings. Furthermore, NaCl stress tolerance is primarily achieved by improved K^+^ uptake and upregulation of non-enzymatic (proline, AsA, GSH) and enzymatic antioxidants (SOD, POX, CAT, APX, and GR). It is also important to note that the K^+^ and H_2_S interplay facilitates the enhanced function of the glyoxalase system, thus leading to the breakdown of MG. As evident from α-amylase, β-amylase, SuSy, SPS, and SAI activity, tomato seedling roots also exhibit higher accumulation of soluble hexose sugar and subsequent osmotic tolerance during K^+^–H_2_S interplay under NaCl stress. Thus, endogenous H_2_S functions as a downstream facilitator of K^+^-mediated alleviation of NaCl stress in tomato seedling roots.

## Figures and Tables

**Table 1:** Effect of potassium and NaCl on oxidative stress markers and related damage. Hydrogen peroxide (H_2_O_2_, n mol g^−1^ DW) content, superoxide (O_2_^•−^, n mol g^−1^ DW) content, thiobarbituric acid reactive substances (TBARSs, n mol g^−1^ DW), relative water content (RWC, %), and electrolyte leakage (ELKG, %).

Treatments	Parameters				
	H_2_O_2_ Content	O_2_^•−^ Content	TBARS	RWC	ELKG
Control	77.38 ± 8.32 de	8.47 ± 1.22 e	12.38 ± 2.61 e	79.42 ± 5.32 ab	8.72 ± 1.56 e
K^+^	58.71 ± 2.76 f	5.32 ± 0.64 f	8.71 ± 0.74 f	83.69 ± 2.61 a	8.61 ± 0.79 ef
K^+^+HT	94.21 ± 1.98 c	11.74 ± 2.67 c	18.32 ± 2.18 c	58.71 ± 1.31 d	14.38 ± 1.92 c
NaCl	136.07 ± 4.51 b	18.36 ± 1.29 b	24.54 ± 1.29 b	36.82 ± 1.97 e	22.71 ± 2.05 b
NaCl+K^+^	79.47 ± 3.92 d	10.61 ± 0.88 cd	14.39 ± 0.76 d	77.91 ± 3.02 bc	10.66 ± 0.98 d
NaCl+K^+^+HT	168.29 ± 4.71 a	21.71 ± 1.38 a	28.71 ± 1.38 a	26.52 ± 2.57 f	29.85 ± 1.77 a

Values are means ± SE of five independent replicates. Values with different letters within a column indicate that differences were statistically significant at *p* < 0.05 (DMRT). Double deionized water (control), 5 mM K_2_CO_3_ (K^+^), 100 mM NaCl (NaCl), 1 mM hypotaurine (HT: H_2_S scavenger).

**Table 2:** Effect of potassium (K^+^) and NaCl on the components of glyoxalase system and proline content. Activity of glyoxalase I/II (Gly I/Gly II, nmol min^−1^ mg^−1^ protein), methylglyoxal (MG, µmol g^−1^ FW) content, and proline (Pro, mg g^−1^ DW) content.

Treatments	Parameters			
	Gly I Activity	Gly II Activity	MG Content	Pro Content
Control	0.128 ± 0.014 d	0.147 ± 0.031 d	12.54 ± 1.32 de	10.98 ± 0.765 cd
K^+^	0.149 ± 0.049 c	0.328 ± 0.004 c	9.65 ± 2.71 f	11.62 ± 0.849 c
K^+^+HT	0.115 ± 0.007 ef	0.109 ± 0.012 e	15.74 ± 1.32 c	6.54 ± 0.654 f
NaCl	0.374 ± 0.018 b	0.401 ± 0.005 b	23.72 ± 0.98 b	15.73 ± 1.227 b
NaCl+K^+^	0.481 ± 0.010 a	0.582 ± 0.017 a	13.52 ± 1.02 d	18.28 ± 1.678 a
NaCl+K^+^+HT	0.117 ± 0.011 e	0.091 ± 0.009 f	27.41 ± 2.61 a	7.36 ± 0.578 e

Values are means ± SE of five independent replicates. Values with different letters within a column indicate that differences were statistically significant at *p* < 0.05 (DMRT). Double deionized water (control), 5 mM K_2_CO_3_ (K^+^), 100 mM NaCl (NaCl), 1 mM hypotaurine (HT: H_2_S scavenger).

## Data Availability

Not applicable.
